# Prevalence of KIG-grades 3–5 in an orthodontic practice in North Rhine Westphalia compared with results of the DMS•6 and with KZBV data

**DOI:** 10.1186/s13005-023-00402-0

**Published:** 2024-01-04

**Authors:** Gero Stefan Michael Kinzinger, Jan Hourfar, Jörg Alexander Lisson

**Affiliations:** Department of Orthodontics, Saarland University, 66424 Homburg, Saar Germany

**Keywords:** Orthodontics, Care, KIG-classification, KIG-grade, DMS•6, Regionality

## Abstract

**Background and aim:**

The prevalence of tooth and jaw malocclusions in 8- to 9-year-olds was surveyed in a nationwide setting as part of the orthodontic module of the Sixth German Study on Oral Health (DMS•6), using the orthodontic indication groups (KIG) as index. Aim of this study was the detection of the prevalence of malocclusions requiring treatment according to the KIG index in statutorily insured patients of an orthodontic practice in North Rhine Westphalia, Germany, and to compare results with corresponding DMS•6 and KZBV data.

**Patients and methods:**

Between 2017–2021, *n* = 953 statutorily insured patients called for an initial consultation and subsequent determination of the KIG-classification and -grades. The malocclusions were classified and graded in the highest possible KIG-grade according to valid SHI guidelines. Multiple classifications were not recorded. KIG-grade > 3 according to the valid guidelines was detected in *n* = 815 patients. Since the DMS•6 does not contain information on KIG classifications "U" and "S", their inclusion was waived despite evaluation, leaving data from *n* = 683 patients for analysis and comparison.

**Results:**

During the study period, *n* = 235 patients (34.4%) had KIG-classification "D". More than 10% were classified as "K" (120 patients, 17.6%), "P" (98 patients, 14.2%), "M" (89 patients, 13.0%), and "E" (81 patients, 11.9%). Of 16 possible classifications with KIG-grade > 3, "D4" was the most common with 26.6% (182 patients). The results confirm the findings from the multicentric DMS•6 from2021 and corresponding KZBV data from 2020.

**Conclusions:**

Sagittal deviations described by classifications "D" and "M" represent with 47.4% almost half of the malocclusions with treatment need. KIG-grade D4 is the most frequent classification. There were no regional deviations of the prevalence of KIG-grades 3–5 in the district of Viersen / North Rhine compared with the national average, not even when scrutinizing a five-year-period.

## Introduction

Orthodontics aim at the detection, prevention, and treatment of malformations of the masticatory system, tooth position and bite anomalies, jaw malformations and deformations of the jaws and the facial skull. The economic efficiency principle in Germany means that not all medically indicated treatments can be considered. The assumption of costs for orthodontic treatment was restricted within the framework of the statutory health insurance (GKV;»Gesetzliche Krankenversicherung«) on 01.01.2002 by the introduction of the orthodontic indication groups (KIG;»Kieferorthopädische Indikationsguppen«) (Table 1) [1]. Orthodontists must determine the patient's treatment need using KIG-classifications during the initial examination. According to current social legislation (paragraph §29 1 SGB V (»Sozialgesetzbuch« (SGB)»fünf« (V)), statutorily insured patients are only entitled to orthodontic care if they have malocclusions of a certain degree of expression or severity (KIG-grades 3–5), where it can be assumed that chewing, biting, speaking, or breathing is or threatens to become significantly impaired [2].

**Table 1:** Orthodontic indication groups (KIG) according to the guidelines of the federal committee of dentists and health insurance funds for orthodontic treatment (figures in mm, “- “ = not applicable)

KIG	Description	Grade 1	Grade 2	Grade 3	Grade 4	Grade 5
A	Craniofacial anomalies	-	-	-	-	(Cleft palate and syndromes)
U	Missing teeth (Agenesis or loss)	-	-	-	missing teeth	-
S	Disturbance in tooth eruption	-	-	-	impaction (except for third molars)	displacement (except for third molars)
D	Sagittal discrepancy increased overjet	≤ 3	> 3, ≤ 6	-	> 6, ≤ 9	> 9
M	Sagittal discrepancy negative overjet	-	-	-	0, ≤ 3	> 3
O	Vertical discrepancy open bite	≤ 1	> 1, ≤ 2	> 2, ≤ 4	> 4 habitually open	> 4 skeletally open
T	Vertical discrepancy deep bite	> 1, ≤ 3	> 3 with / without mucosal contact	> 3 with traumatic mucosal impingement	-	-
B	Transverse discrepancy scissors bite	-	-	-	Scissors bite	-
K	Transverse discrepancy crossbite	-	Buccolingually cusp-to-cusp relation	Bilateral crossbite	unilateral crossbite	-
E	Contact point displacement	< 1	> 1, ≤ 3	> 3, ≤ 5	> 5	-
P	Space deficiency	-	≤ 3	> 3, ≤ 4	> 4	-

As part of the current Sixth German Study on Oral Health (DMS•6), a validated and representative epidemiological survey was conducted in the KFO-6.1 module regarding the nationwide prevalence of dental and jaw malocclusions in 8- to 9-year-olds. These results were first presented at the annual convention of the German Orthodontic Society (DGKFO) in 2022 and subsequently published in the Journal of Orofacial Orthopedics in 2023 [3–6]. The primary objective of this study was to record the prevalence of malocclusions in 8- and 9-year-old children in Germany, and to derive the need for orthodontic care as a secondary objective. Data were collected between January and March 2021 in 16 locations in Germany, representative for each federal state. All relevant data were available for statistical analysis from *n* = 705 study participants born in 2011 and 2012 (51.4% m, 48.6% f). The proportion of 8-year-olds was 49.4%, that of 9-year-olds 50.6%. The KIG-classifications "U" (aplasia) and "S" (eruption disorders) were not recorded in the DMS•6, as no x-rays were available. Multiple responses were possible for the remaining 9 KIG-classifications. Orthodontic treatment was indicated in *n* = 286 study participants according to the current guidelines of the statutory health insurance (KIG-grades 3–5), which corresponds to a rate of 40.4%.

In the DMS•6, National Association of Statutory Health Insurance Dentists (KZBV) billing data from 2020 were also published [7], including the KIG-grades 3–5 of all age groups. However, it is still unclear if these cross-section, nationwide results correspond to those of a regional population recorded long-term.

### Aims of the study

The aims of this study were:

1. to determine the prevalence and severity of KIG-classifications (KIG-grades 3–5) requiring treatment in an orthodontic practice from North Rhine Westphalia, Germany, in statutorily insured patients over a five-year period between 2017 and 2021,
2. to determine the distribution of KIG-grades 3–5 among those patients, and
3. to compare the results from this five-year period with results of epidemiologic data from DMS•6 and KZBV.

## Methods

### Patient acquisition

An unselected cohort of patients with statutory health insurance was drawn from an orthodontic practice in the District of Viersen, North Rhine Westphalia, Germany. Over a five-year period between 2017 and 2021, KIG-classifications and -grades were collected and documented for *n* = 953 patients during their initial consultation. The period was chosen to overlap the data collection phase of the DMS•6.

Several KIG-classifications can trigger combined orthodontic treatment with orthognathic surgery in adults. Since this is a rare occurrence, only few patients aged 18 and above could be included (*n*=10). 

### Data acquisition

Tooth and jaw malposition were recorded in all possible classifications of the KIG system (Table 1). The KIG-classifications "U" (aplasia) and "S" (ectopy and retention) were recorded but not listed. Since the DMS•6 does not contain information on KIG-classifications "U" and "S", their inclusion was waived despite evaluation, leaving data from *n* = 683 patients for analysis and comparison.

The diagnoses were primarily obtained through clinical inspection, as required by legislation. The extent and direction of overjet, and overbite, anterior crowding and space deficits were measured intraorally using sliding calipers»Münchner Modell®« (Dentaurum, Ispringen, Germany) with a precision of 0.25 mm. The assessment of occlusion regarding frontal and lateral crossbites was performed visually. Only if justified by clinical reasons, x-rays were made to detect possible aplasia, ectopy or retention of permanent teeth. Children and adolescents up to the age of 18 as well as adult patients who required orthognathic surgery were analysed. The classification of the data sets was carried out according to the valid framework of the guidelines of the Statutory Health Insurance (GKV) [8]. This means that even if several KIG-grades > 3 were present, they were categorized exclusively according to the highest possible classification and grade.

Within the framework of the DMS•6, extrapolated billing data of the National Association of Statutory Health Insurance Dentists (KZBV) were presented as comparative figures [7]. The DMS•6 thus served as an indirect source for the figures used.

For comparison with the results of the DMS•6 and the KZBV billing data, only KIG-grades requiring treatment (KIG-grades 3–5) were recorded.

### Statistics

Anonymized patient data was collated using a spread sheet software (Excel®, Microsoft Corp., Redmond, WA, USA). Normal distribution of the variable “age” was evaluated graphically and using the Shapiro–Wilk-Test with SPSS® Version 28 for Windows® (IBM Corp., Armonk, NY, USA). Mean and standard deviation was recorded. All other data were interpreted descriptively.

## Results

### Patients (Fig. 1, Table 2)

**Fig. 1:**
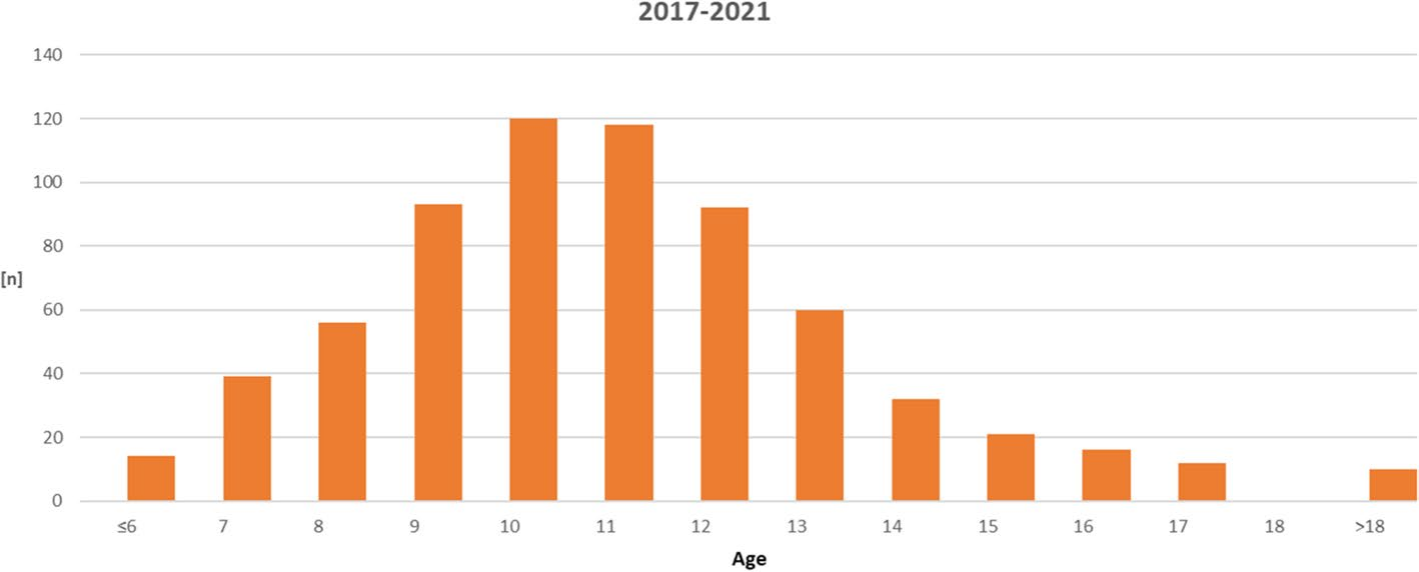
Age distribution of *n* = 683 statutorily insured patients between 2017 and 2021 at initial orthodontic consultation with KIG-grades 3–5

**Table 2:** Age and gender distribution of *n* = 683 statutorily insured patients between 2017 and 2021 at initial orthodontic consultation and KIG-grades 3, 4 and 5 between 2017 and 2021

Patient distribution n (%)			Patient age [yrs]			Number of patients in category “age” [n]													
**total**	**female**	**male**	**M**	**Min**	**Max**	** ≤ 6**	**7**	**8**	**9**	**10**	**11**	**12**	**13**	**14**	**15**	**16**	**17**	**18**	** > 18**
683 (100.00)	365 (53.4)	318 (46.6)	11.5	3.6	44.1	14	39	56	93	120	118	92	60	32	21	16	12	0	10

*N* = 815 out of *n* = 953 statutorily insured patients required orthodontic treatment according to the applicable guidelines. As the KIG classifications "U" (aplasia, *n* = 46 patients) and "S" (eruption disorders, *n* = 86 patients) were omitted due to the methodology, *n* = 683 patients with an age peak at 10 and 11 years remained to be analysed. The patient age distribution is shown in Fig. 1 and Table 2.

### Prevalence of KIG-classifications (Fig. 2, Table 3)

**Fig. 2:**
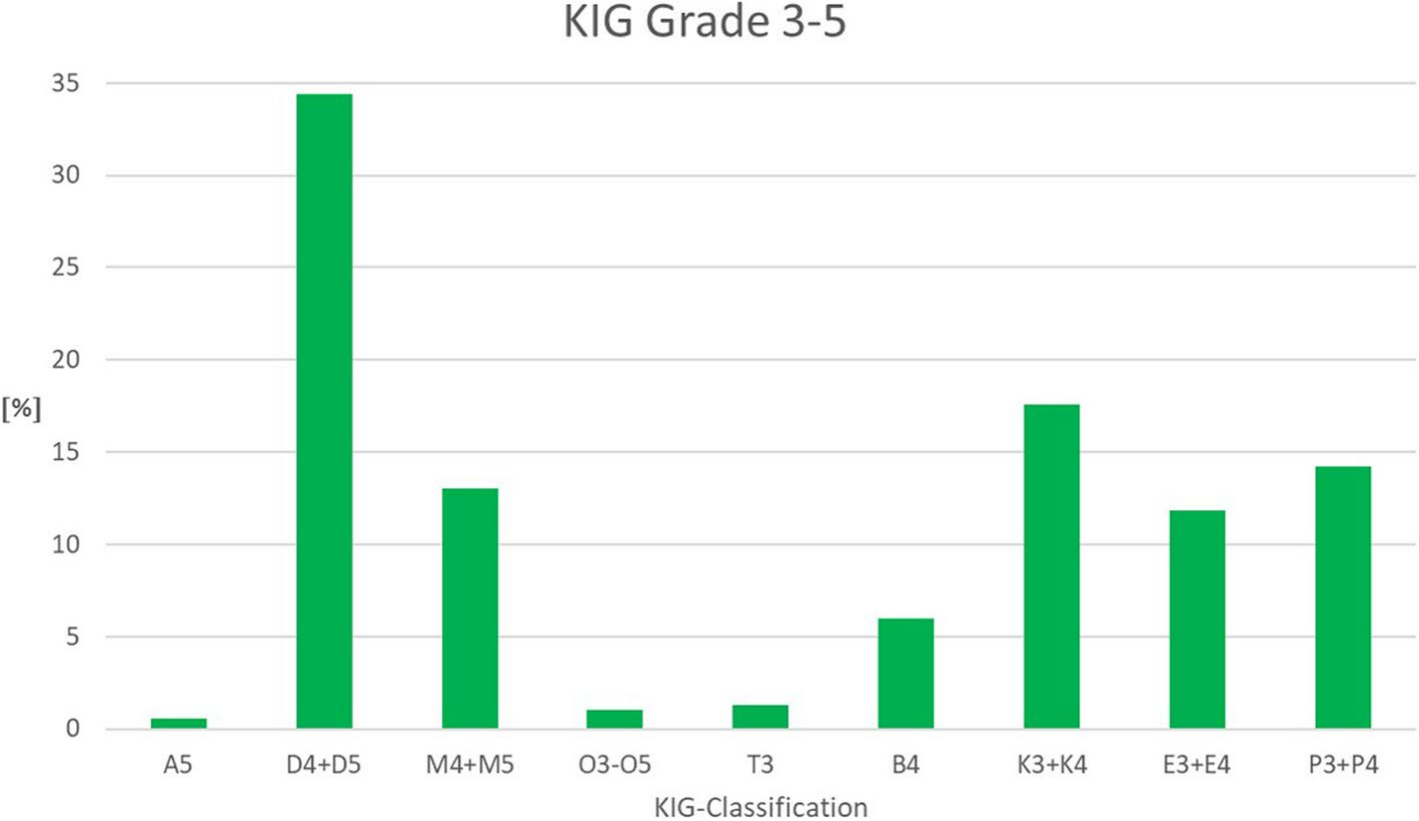
Percentage of the 9 different KIG-classification groups among patients with statutory health insurance in the observed period 2017–2021

**Table 3:** Percentage distribution of different KIG-grades requiring treatment (9 classifications and 16 grades) in patients with statutory health insurance between 2017 and 2021

KIG	Description	Grade 3 [n]	Grade 3 [%]	Grade 4 [n]	Grade 4 [%]	Grade 5 [n]	Grade 5 [%]	Grade 3—5 [n]	Grade 3—5 [%]
A	Craniofacial anomalies	-	-	-	-	4	0.6	4	0.6
D	Sagittal discrepancy increased overjet	-	-	182	26.6	53	7.8	235	34.4
M	Sagittal discrepancy negative overjet	-	-	83	12.1	6	0.9	89	13.0
O	Vertical discrepancy open bite	4	0.6	2	0.3	1	0.1	7	1.0
T	Vertical discrepancy deep bite	9	1.3	-	-	-	-	9	1.3
B	Transverse discrepancy scissors bite	-	-	41	6.0	-	-	41	6.0
K	Transverse discrepancy crossbite	9	1.3	111	16.3	-	-	120	17.6
E	Contact point displacement	77	11.3	4	0.6	-	-	81	11.9
P	Space deficiency	38	5.6	59	8.6	-	-	97	14.2
**total**		**137**	**20.1**	**482**	**70.5**	**64**	**9.4**	**683**	**100.0**

Within the observed five-year-period, *n* = 235 (34.4%) had the KIG-classification "D".

The KIG-classifications "K" (120 patients, 17.6%), "P" (97 patients, 14.2%), "M" (89 patients, 13.0%), and "E" (81 patients, 11.9%) each accounted for more than 10%.

The KIG-classifications "B" (41 patients, 6.0%) each represented more than 5%, "T" (9 patients, 1.3%), "O" (7 patients, 1.0%) and "A" (4 patients, 0.6%) less than 2%.

Of 9 of the 11 possible classifications, a total of 97.1% were assigned to the 6 most frequent classifications ("D", "K", "P", "M", "E" and "B") and 2.9% to the 3 rarest classifications ("T", "O" and "A").

### Frequency of eligible KIG-grades 3–5 (Fig. 3, Table 3)

**Fig. 3:**
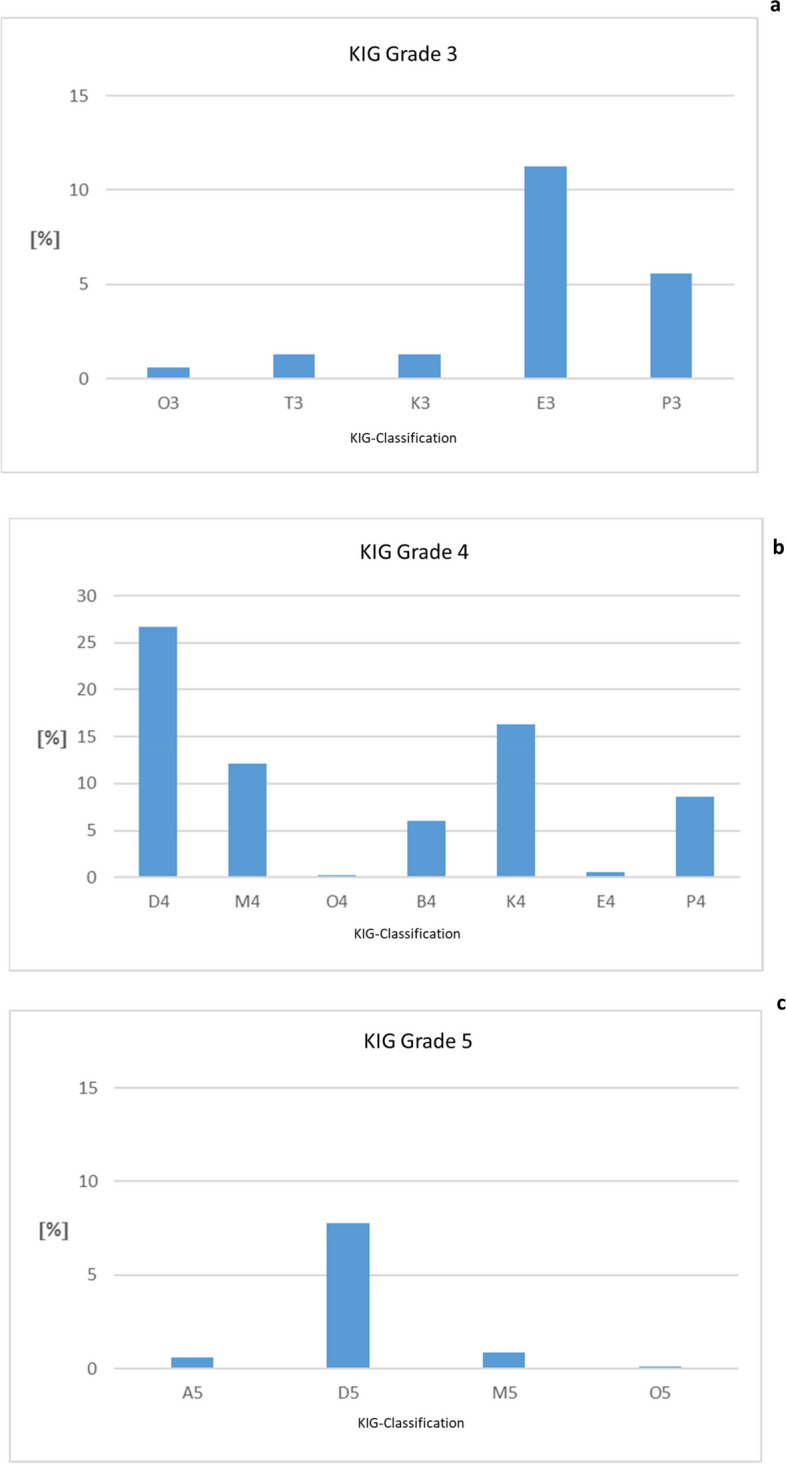
**a**-**c**: Percentage of the 16 possible KIG-grades triggering treatment for patients with statutory health insurance in the observed period 2017–2021. **a** KIG-Grade 3. **b** KIG-Grade 4. **c** KIG-Grade 5

Of the 16 possible classifications eligible for treatment, "D4" was most the most common with 26.6% (182 patients). More than 10% each were distributed among the KIG-grades "K4" (111 patients, 16.3%), "M4" (83 patients, 12.1%) and "E3" (77 patients, 11.3%), and more than 5% among "P4" (59 patients, 8.6%), "D5" (53 patients, 7.8%), "B4" (41 patients, 6.0%) and "P3" (38 patients, 5.6%). The 8 most frequent KIG-grades thus have a share of 94.3%.

Of *n* = 683 patients, 20.1% had pronounced malocclusions (KIG-grade 3), 70.5% had very pronounced malocclusions (KIG-grade 4) and 9.4% had extremely pronounced malocclusions (KIG-grade 5).

### Subdivision according to spatial plane and tooth malposition

- The prevalence of sagittal anomalies "D" and "M" is 47.4%,
- The prevalence of transverse anomalies "B" and "K" is 23.6%
- The prevalence of vertical anomalies "O" and "T" is 2.3%.
- The prevalence of tooth malposition "E" and "P" is 26.1%.

### Synopsis of DMS•6 data (Table 4)

**Table 4:** Percentage distribution of different KIG-grades requiring treatment (9 classifications and 16 grades) of the DMS•6, sample of 8- and 9-year-olds (Source: [7] p 84, tab. 3.25)

KIG	Description	Grade 3	Grade 4	Grade 5	Grade 3—5
A	Craniofacial anomalies	-	-	1.2	1.2
D	Sagittal discrepancy increased overjet	-	37.8	8.1	45.9
M	Sagittal discrepancy negative overjet	-	6.9	1.5	8.5
O	Vertical discrepancy open bite	1.5	-	2.3	3.9
T	Vertical discrepancy deep bite	12.4	-	-	12.4
B	Transverse discrepancy scissors bite	-	0.4	-	0.4
K	Transverse discrepancy crossbite	-	9.7	-	9.7
E	Contact point displacement	6.6	-	-	6.6
P	Space deficiency	5.4	6.2	-	11.6
**total**		**25.9**	**61.0**	**13.1**	**100.0**

Of the DMS•6 [7] patients aged 8 and 9 years, *n* = 286 had a KIG-grade 3–5 and were therefore eligible for orthodontic treatment according to the current legislation (Table 5). 45.9% had the KIG-classification "D". The KIG-classifications "T" (12.4%) and "P" (11.6%) each accounted for more than 10%, and the KIG-classifications "K" (9.7%), "M" (8.5%) and "E" (6.6%) each accounted for more than 5%.

Of 9 of the 11 possible classifications, a total of 94.7% were assigned to the 6 most frequent findings ("D", "T", "P", "K", "M" and "E") and 5.5% to the 3 rarest findings ("O", "A" and "B"). Of the 16 possible classifications eligible for treatment (KIG-grades 3–5), "D4" was the most common at 37.8% (182 patients). Only KIG-grade "T3" (12.4%) showed more than 10%.

Of the *n* = 286 subjects who needed treatment, 10.0% had pronounced malocclusions (KIG-grade 3), 25.5% had very pronounced malocclusions (KIG-grade 4) and 5.0% had extremely pronounced malocclusions (KIG-grade 5).

Broken down by spatial plane and tooth malposition, sagittal deviations "D" and "M" taken together have a share of 54.4%, the transverse deviations "B" and "K" taken together have a share of 10.1%, the vertical deviations "O" and "T" taken together have a share of 16.3%, and "E" and "P" taken together have a share of 18.2%.

### Synopsis of KZBV data (Table 5)

**Table 5:** Percentage distribution of different KIG-grades requiring treatment (9 classifications and 16 grades) in the billing data of the National Association of Statutory Health Insurance Dentists (KZBV) including all age groups in the year 2020 (Source: [7] p 85, tab. 3.26)

KIG	Description	Grade 3	Grade 4	Grade 5	Grade 3—5
A	Craniofacial anomalies	-	-	0.3	0.3
D	Sagittal discrepancy increased overjet	-	26.2	7.5	33.7
M	Sagittal discrepancy negative overjet	-	17.9	0.7	18.6
O	Vertical discrepancy open bite	0.8	0.2	0.3	1.3
T	Vertical discrepancy deep bite	1.5	-	-	1.5
B	Transverse discrepancy scissors bite	-	5.4	-	5.4
K	Transverse discrepancy crossbite	1.8	14.8	-	16.6
E	Contact point displacement	9.3	0.8	-	10.1
P	Space deficiency	6.4	6.0	-	12.4
**total**		**19.9**	**71.4**	**8.7**	**100.0**

As part of the DMS•6 [7], billing data from the National Association of Statutory Health Insurance Dentists (KZBV) on the distribution of the various KIG-classifications eligible for treatment (9 classifications and 16 grades) across all age groups from 2020 were published and used for comparison (Table 4). Nationwide, 33.7% of patients had the KIG-classification "D". The KIG-classifications "M" (18.6%), "K" (16.6%), "P" (12.4%) and "E" (10.1%) each accounted for more than 10%. The KIG-classifications "B" (5.4%) and "A" (0.3%) were the less frequent. Of 9 of the 11 possible classifications, a total of 96.8% were assigned to the 6 most frequent findings ("D", "M", "K", "P", "E" and "B") and 3.1% to the 3 rarest findings ("T", "O" and "A").

Of the 16 possible classifications eligible for treatment (KIG-grades 3–5), "D4" was the most common at 26.2%. The KIG-grades "M4" (17.9%) and "K4" (14.8%) each accounted for more than 10%.

The nationwide data showed that 19.9% of patients with treatment need had pronounced malocclusions (KIG-grade 3), 71.4% had very pronounced malocclusions (KIG-grade 4) and 8.7% had extremely pronounced malocclusions (KIG-grade 5).

Broken down by spatial plane and tooth malposition, sagittal deviations "D" and "M" taken together have a share of 52.3%, the transverse deviations "B" and "K" taken together have a share of 22.0%, the vertical deviations "O" and "T" taken together have a share of only 2.8%, and "E" and "P" taken together have a share of 22.5%.

A synoptic comparison of the present study results with those of DMS•6 and KZBV is shown in Table 6.

**Table 6:** Synoptic comparison of study results with those of DMS•6 and KZBV

Subdivision according to spatial plane and tooth malposition	Present investigation [%]	DMS 6 [%]	KZBV [%]
D + M (sagittal)	47.4	54.4	52.3
O + T (vertical)	2.3	16.3	2.8
B + K (transverse)	23.6	10.1	22.0
E + P (tooth malposition)	26.1	18.2	22.5

## Discussion

### Limitations of the methodology

An intentional limitation of the study is that not all KIG-classifications were recorded. For better comparability, "U" and "S" were excluded from further comparison with DMS•6 and KZBV data, since they are missing in these studies. Studies from other authors show that a prevalence of approximately 5% [9] must be assumed for aplasia (classification "U") and around 6% [10] for ectopy and retention (classification "S"). Within the scope of our own study, a larger proportion with KIG classification "S" (10.5%) was found, while "U" was comparable (5.6%).

A possible study limitation could be that the age of the analyzed group was not adapted to the available data of theDMS•6. No age matching subgroups were formed in the present study, as it can be assumed that the KIG distribution would have been distorted due to the pre-selection of patients by the regular dental practices.

Another possible limitation of the methodology could be that KIG-classifications and -grades were recorded by different examiners within one practice. According to Gesch et al. [11], there are considerable inter-examiner differences in the classification of subjects into the respective indication groups, and thus also different classifications into KIG-grades ≤ 2 and ≥ 3. Different data collection methods (clinic/dental cast) in the assessment of a malocclusion by different or orthodontically inexperienced examiners may have an unfavorable influence on examiner agreement. For this reason, KIG-classifications were made according to the four-eye principle without exception. Especially in borderline cases between KIG-grades ≤ 2 and ≥ 3, classifications were made based on a dental cast and, if necessary, a panoramic x-ray.

### Comparison with available results

The DMS•6 was designed as a baseline study to provide data that will be used for an intra-cohort comparison during the DMS•7 among the same patients at a later age. The DMS•6 patients were randomly picked, while the present study used preselected patients. The selection took place in regular dental practices where malocclusions were detected to a certain degree and sent to the orthodontic specialist for further consideration. Despite the obvious inhomogeneity of the groups, the outcome was surprisingly constant. This allows the deduction that data gained so far are usable for future orthodontic caseload estimates among the German population. When comparing present results with those of the DMS•6 and the KZBV, similarities and differences become apparent. Among all studies, the sagittal classifications "D" and "M" always describe approximately half of all malocclusions requiring treatment, and the KIG-grade "D4" is the most common. However, the KIG-grade "T3" and thus the combined vertical deviations "T" and "O" occur much more frequently in the DMS•6. One possible explanation for this may be that the DMS•6 allowed to record several different KIG-grades ≥ 3 per proband. This means that, unlike in the present study, not only the highest possible KIG-classification and -grade was recorded, but KIG-grades were recorded and counted for each indication that occurred: One proband could have several KIG-grades ≥ 3. This may have led to an overrepresentation of certain KIG-grades, such as “T3” in this case. If the recording procedure is carried out according to the current legal situation with only one classification per patient, KIG-grades 4 or 5 are likely to occur much more frequently.

The comparison of present results with the KZBV billing data shows a high degree of consistency in all areas. Accordingly, there are no regional deviations from the national average. Both in terms of age distribution and frequency of findings, the results obtained in North Rhine Westphalia correspond to those of the KZBV.

It should be noted that the KIG-classification represents a control instrument within the framework of social legislation to restrict access to treatments at the expense of the GKV framework. This determines that – with certain exceptions – regular treatment is not provided for patients before the late mixed dentition, i.e., approximately from the age of 10. The application of the KIG-classification to 8- and 9-year-olds as in the DMS•6 is thus not uncritical, as orthodontic anomalies become more pronounced with increasing age, especially during pubertal growth [12, 13]. However, the focus on younger probands in the DMS•6 was deliberate to avoid a possible bias due to early orthodontic treatment, which is often carried out before the age of 10.

## Conclusions

The present five-year unicentric cross-sectional study confirms the results of the one-time multicentric DMS•6 from 2021 and multicentric KZBV data from 2020. The combination of sagittal deviations "D" and "M" always accounts for approximately half of all classifications triggering treatment and KIG-grade "D4" (Overjet > 6 mm) occurs most common. Regional deviations from this are also not recognisable in the Viersen / North Rhine Westphalia district. The prevalence and age distribution of KIG-grades 3–5 requiring treatment is consistently in line with the national average.

## Data Availability

Not applicable.
